# Editorial: New insights into presynaptic G protein-coupled receptors and addiction

**DOI:** 10.3389/fncel.2023.1358243

**Published:** 2024-01-08

**Authors:** Massimo Grilli

**Affiliations:** Department of Pharmacy, School of Medical and Pharmaceutical Sciences, University of Genoa, Genoa, Italy

**Keywords:** BDNF, G coupled-protein receptor, addiction, glutamate, acetycholine, GABA, noradrenaline (NA), volume transmission

Metabotropic receptors are widely recognized as pharmacological targets for various brain diseases (Nicoletti et al., 1996, 2011; Bruno et al., 2001). Sophisticated optical techniques have advanced the examination of receptors and G proteins within living cells. These investigations have revealed a previously unknown level of complexity, demonstrating that G protein-coupled receptors (GPCRs) engage in brief interactions with each other, as well as with G proteins and structural components of the cell membrane, to create short-lived signaling nanodomains. These nanodomains are highly dynamic and ephemeral, allowing for rapid and specific signaling events to occur (Calebiro et al., 2021). The phenomenon of addiction has also been extensively studied, and a significant amount of evidence has emerged regarding the role of GPCRs (Koob and Volkow, 2010; Cleva and Olive, 2012). Their pre- and post-synaptic localization has stimulated scientific analysis of their involvement in numerous neurological processes. Metabotropic receptors are expressed not only on the neuronal component of the active milieu but also on virtually all the elements that compose it (Semyanov and Verkhratsky, 2021, 2022). Immune cells, such as microglia and macrophages, express metabotropic receptors that play a role in regulating the immune response, including cytokine release and T-cell activation. The interest in addiction is justified by its social impact, the development of comorbidities and the continuous evolution of psychoactive substances. It is equally true that new forms of addiction, such as cyber addiction are emerging. The problem affects people of all ages with different characteristics, globally. Often, to analyze this level of complexity, researchers have started from the common ground of addiction, which specifically affects the brain areas involved. In particular, the limbic area has been widely characterized for its expression in metabotropic receptors.

Several neuromodulatory systems involved in addiction-related behavior activate presynaptic GPCRs at glutamatergic synapses in the nucleus accumbens (NAc). However, the specific pathways through which these receptors affect glutamatergic synapses in the NAc are poorly understood. In this regard, Manz et al. identified GPCR systems which mediate the depression of EPSCs through the SNAP25-Gβγ interaction. Utilizing patch-clamp electrophysiology in a genetically modified mouse line with a three-residue deletion at the C-terminus of SNAP25, a modification that weakens the interaction between Gβγ and SNARE proteins, researchers unraveled the distinct interactions of GABAB, 5-HT1B/D, and μ opioid heteroreceptors with SNAP25. In contrast, κ opioid, CB1, adenosine A1, group II metabotropic glutamate, and histamine H3 receptors consistently blocked glutamatergic transmission onto medium spiny projection neurons, in a SNAP25 independent manner.

Understanding the effects of exogenous cannabinoids also requires understanding the mechanisms used by endogenous modulators. In this regard, Chlieh and Levine present a study on the effects of 2-arachidonylglycerol (2-AG) and anandamide on hippocampal Long-Term Potentiation (LTP). The researchers found that electrical and pharmacological (forskolin plus rolipram) LTP stimulation was helped by endogenous cannabinoid release. This cooperative mechanism reduced the GABAergic negative modulation through the simultaneous activation of CB1 receptors.

The locus coeruleus (LC) is a small, almond-shaped cluster of neurons located within the brainstem. As the main source of norepinephrine, a neurotransmitter associated with numerous functions including alertness, mood and stress response, the LC is believed to play a crucial role in addiction. Research has established that individuals with addiction exhibit elevated levels of norepinephrine in their LCs, indicating the potential involvement of the LC in both the onset and persistence of addiction. Toyoda et al. conducted a study on the volume transmission of Noradrenaline from the Locus Coeruleus (LC) to the neurons of the mesencephalic trigeminal nucleus, which is accompanied by α2A-adrenergic GPCR activation. This transmission can be characterized by three distinct patterns of inhibition in response to three different patterns of repetitive activation of LC neurons.

The full extent of addiction's impact on various body organs remains incompletely elucidated. For example, in nicotine and ketamine addiction some studies discussed a loss of kidney function. Yeung et al. (2009) propose that chronic ketamine addiction could lead to the degeneration of neuromuscular junctions and/or proprioceptive sensory fibers. Bogacheva et al. highlight the role of endogenous brain-derived neurotrophic factor (BDNF), its precursor, and its mature form in maintaining the homeostasis of acetylcholine neurotransmission at neuromuscular junctions. The inhibitory effects of these two brain-derived neurotrophic factor-related proteins are mediated through G-protein-coupled inward-rectifying potassium channels.

In this Research Topic, we can identify novel mechanisms involving the glutamatergic, noradrenergic, cholinergic, and cannabinoid systems. These systems have all been extensively connected to the phenomenon of addiction (Figure 1). This suggests a growing complexity that once again implicates metabotropic receptors and the specific cascade of events associated with them as potential pharmacological targets.

**Figure 1:**
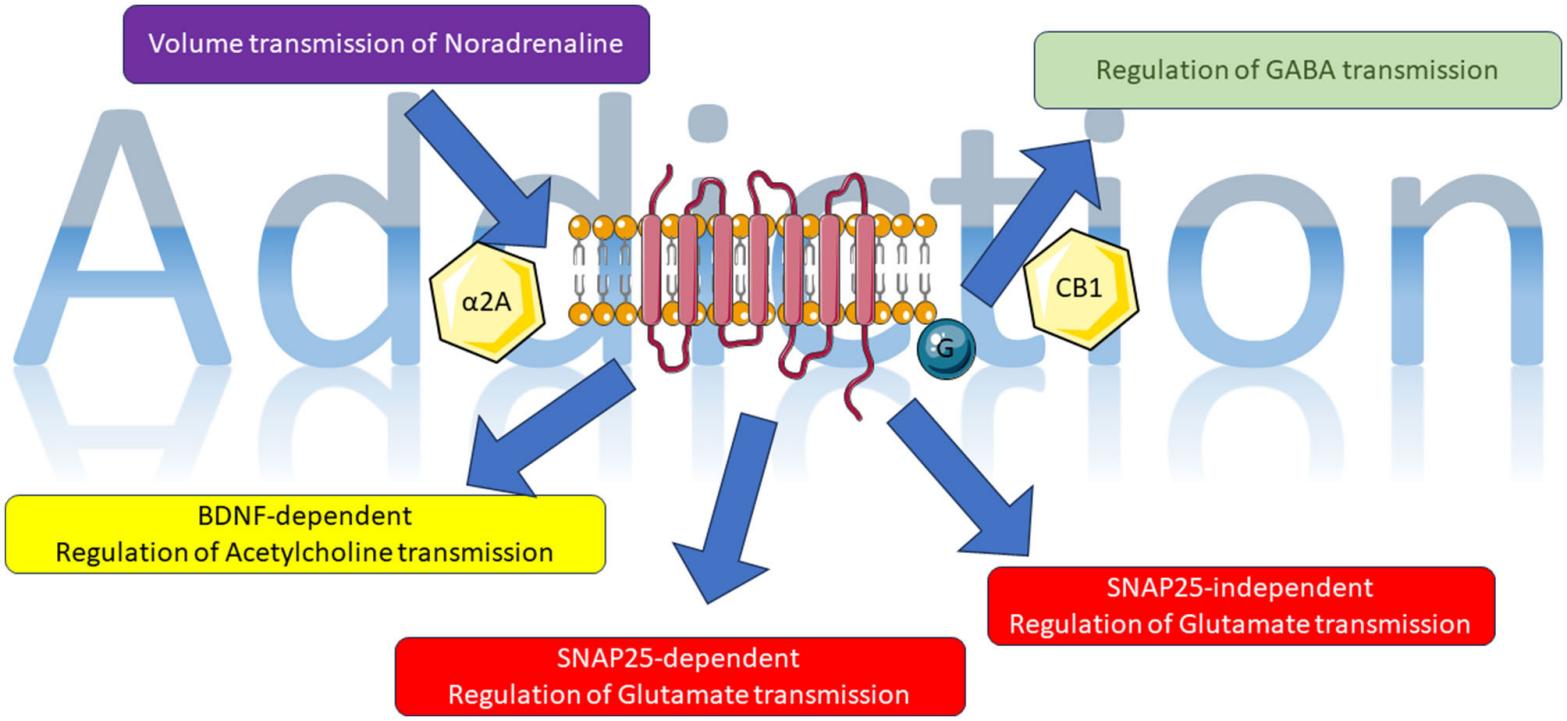
Representative image of data presented in this Research Topic; for details, see main text. The figure was partly generated using Servier Medical Art, provided by Servier, licensed under a Creative Commons Attribution 3.0 unported license (https://smart.servier.com/, accessed on 14 June 2023).

## Author contributions

MG: Conceptualization, Resources, Supervision, Validation, Visualization, Writing – original draft, Writing – review & editing.
